# AMPK blunts chronic heart failure by inhibiting autophagy

**DOI:** 10.1042/BSR20170982

**Published:** 2018-07-18

**Authors:** Yanhui Li, Yan Wang, Man Zou, Cong Chen, Yili Chen, Ruicong Xue, Yugang Dong, Chen Liu

**Affiliations:** 1 Department of Cardiology, The First Affiliated Hospital of Sun Yat-Sen University, Guangzhou 510080, China; 2 Department of Internal Medicine, Institute of Hypertension, Tongji Hospital, Tongji Medical College, Huazhong University of Science and Technology, Wuhan 430030, China; 3 Key Laboratory of Assisted Circulation, Ministry of Health, Guangzhou 510080, China; 4 Department of Heart Failure, the First Affiliated Hospital of Sun Yat-Sen University, Guangzhou 510080, China

**Keywords:** autophagy, AMPK, heart failure, mechanistic target of rapamycin

## Abstract

AMP-activated protein kinase (AMPK), a serine/threonine protein kinase, has been shown to exert a protective effect against cardiac hypertrophy and heart failure. Our previous reports have demonstrated that AMPK can inhibit cardiac hypertrophy and block the development of heart failure by promoting autophagy. However, other investigators have demonstrated that overactive and dysregulated autophagy may also contribute to the onset and exacerbation of heart failure. Thus, a major goal of the present investigation is to explore how AMPK regulates autophagy in heart failure. First, heart failure was induced in mice by 4 weeks of pressure overload; AMPK activation was subsequently induced by injecting 5-aminoimidazole-4-carboxamide 1-β-d-ribonucleotide (AICAR) after the establishment of chronic heart failure. We showed that AMPK activation significantly attenuated the progression of heart failure and improved cardiac function, which was accompanied by decreased autophagy levels in the failing hearts. Additionally, we demonstrated that the treatment with AICAR inhibited phosphorylation of the mammalian target of rapamycin (mTOR) complex 1 (mTORC1) downstream effectors 4E-binding protein1 (4EBP1), and ribosomal protein S6 kinase (p70S6K). A major action of AICAR was significantly to activate AKT (Ser^473^), the downstream substrate of mTOR complex 2 (mTORC2). In conclusion, the data suggest that AMPK improved cardiac function during the development of chronic heart failure by attenuating autophagy, potentially via mTORC2 activation and the downstream effects.

## Introduction

Cardiac hypertrophy occurs in response to stresses from neurohumoral activation and hemodynamic overload. Persistent cardiac hypertrophy usually progresses to chronic heart failure, which has a high mortality rate. Initially, the heart undergoes adaptive morphological changes that result in compensated hypertrophy to maintain cardiac output. Sustained hypertrophy is irreversible and inevitably results in decompensated heart failure under prolonged stress. Autophagy, a conserved process that degrades dysfunctional proteins and cellular components, has been shown to play intricate roles in cardiac hypertrophy and heart failure [1]. It has been reported that decreased autophagy in tuberous sclerosis complex 2 (TSC2^−/−^) mice leads to cardiac dysfunction and cardiomyocyte hypertrophy, indicating that basal autophagy is crucial for the maintenance of cardiac morphology and function [2]. Moreover, cathepsin-L antagonizes cardiac hypertrophy by facilitating autophagy and proteasomal protein processing [3]. In contrast, multiple studies have suggested that overactive and dysregulated autophagy may play a role in the transition from stable cardiac hypertrophy to decompensated heart failure [4–6]. Indeed, overexpression of beclin-1, a proautophagy protein, stimulates autophagy and exacerbates cardiac dysfunction and pathological remodeling [6]. In addition, evidence has shown that autophagy is activated during heart failure in multiple cardiovascular diseases, such as dilated cardiomyopathy (DCM), ischemic heart disease, and valvular disease [4,5]. Thus, the level of autophagy in the heart may determine whether the autophagy will be protective or deleterious.

Mammalian AMP-activated protein kinase (AMPK) is a serine/threonine protein kinase containing a catalytic α subunit and regulatory β and γ subunits [7]. AMPK exerts protective effects against cardiac hypertrophy and heart failure [7–9]. Our previous studies have demonstrated that AMPK can attenuate cardiac hypertrophy induced by pressure overload or phenylephrine (PE) by blocking various signaling pathways, including nuclear factor of activated T cells (NFAT), MAPK, NF-κB, FOXO, and MuRF1 [10–12]. Moreover, AMPK activators (metformin and 5-aminoimidazole-4-carboxamide 1-β-d-ribonucleotide (AICAR)) restore cardiac function in failing hearts induced by rapid ventricular pacing or coronary occlusion [8,9].

AMPK has shown to be an important modulator of autophagy. Suppression of calcium-sensing receptors can attenuate isoproterenol-induced cardiac hypertrophy, which is associated with the inhibition of autophagy and down-regulation of CaMKK-β-AMPK signaling [13]. In addition, enhanced autophagy via AMPK signaling is an adaptive response to inhibit Epac1-induced cardiomyocyte hypertrophy [14]. Moreover, recent evidence has demonstrated that impaired autophagy due to the down-regulation of AMPK signaling underlies cardiac hypertrophy induced by chronic intermittent hypoxia [15]. We previously showed that induction of autophagy by AMPK activation could inhibit cardiac hypertrophy, thereby blocking the development of heart failure by promoting autophagy [10]. However, dysregulated autophagy may also attribute to the development of heart failure [4–6]. Given the proautophagic effect of AMPK, whether AMPK can protect against cardiac dysfunction in heart failure by regulating autophagy remains unknown. Thus, in the present study, we first induced heart failure by pressure overload and then activated AMPK in the failing hearts to further illustrate how AMPK regulates autophagy in heart failure and to explore the potential underlying mechanisms.

## Materials and methods

### Reagents

Antibodies were obtained from the following companies: GAPDH, beclin-1, pho-ribosomal protein S6 kinase (p70S6k) (T389), p70S6k, pho-4E-binding protein1 (4EBP1) (T37/46), 4EBP1, pho-AKT (Ser^308^), pho-AKT (Ser^473^), and AKT (Cell Signaling Technology, Beverly, U.S.A.); LC3B (Novus, Colorado, U.S.A.); and α-actin and p62 (Sigma, MO, U.S.A.). F-12 medium (high glucose) and collagenase were purchased from Gibco BRL (Grand Island, NY, U.S.A.), and FBS was from Nego (Shanghai, China). Cell lysis buffer (10×) was from Cell Signaling Technology (Massachusetts, U.S.A.). AICAR was purchased from Toronto chemicals, and PE was from Tokyo Chemical Industry.

### Animals

All experimental protocols complied with the Guide for the Care and Use of Laboratory Animals published by the U.S. National Institutes of Health and the Animal Care and Use Committees of Sun Yat-Sen University. Eight- to ten-week-old male C57BL/6J mice weighing 24–26 g were used in the current study. Mice were anesthetized by intraperitoneal injection of 1.5% pentobarbital (W*0.06), and heart failure was induced by pressure overload via descending aortic banding (AB) [10]. To study the effect of AMPK on chronic heart failure, the mice were first subjected to AB for 4 weeks in order to induce heart failure; these mice were subsequently treated with intraperitoneal injections of 0.5 mg/g AICAR (AMPK activator) for another 4 weeks. Control mice received an equal volume of normal saline. All mice were anesthetized with 2% isoflurane inhalant before they were killed.

### Echocardiography

Mice were administered inhalant anesthetics (1.5–2% isoflurane inhalant mixed with 1 l/min 100% O_2_) before examination. Transthoracic echocardiography (Visual Sonics Vevo2100 with a 30-MHz transducer) was performed by an experienced technologist who was blinded to the study group.

### Western blotting

Protein was extracted from the cells and left ventricular tissues by cell lysis buffer supplemented with PMSF and protease inhibitors. Protein concentration in cell lysates was measured using a Bio-Rad DC Protein Assay Kit. Western blotting was performed using standard procedures described in previous reports [2,6] and visualized with X-ray film. Signals were quantitated using Quality One software.

### Immunofluorescence confocal microscopy

Heart tissue was embedded in OCT and floated on isopentane cooled with dry ice and then cut into 5-mm sections. The dried cryosections were washed with PBS and then incubated in 0.3% H_2_O_2_ for 30 min. The samples were blocked with 10% BSA for 1 h at room temperature, incubated with primary antibodies, and then washed and incubated with fluorescent secondary antibodies. Finally, cryosections were mounted with Pro-Long Gold Fluorescent Mounting Medium (Invitrogen) and imaged using an LSM 710 confocal microscope system (German Zeiss).

### TEM

Briefly, small tissue samples (approximately 1–2-mm cubes) were fixed by immersion in 2% paraformaldehyde and 2.5% glutaraldehyde for over 4 h and post-fixed for 1 h in 1% OsO_4_ in 0.1 M PBS. The tissues were cut into 60–80-nm sections with an ultramicrotome, placed on TEM grids, stained with lead citrate, and imaged using a TecnaiG [2] spirit Twin transmission electron microscope (FEI, U.S.A.) at 80 kV.

### Statistical analyses

All the data are expressed as the mean ± S.E.M. Differences between the means were evaluated using two-way ANOVA. *P*-values less than 0.05 were considered statistically significant. Statistical analyses were performed using SPSS13.0 software.

## Results

### AMPK inhibited the aggravation of heart failure

To study the effects of AMPK on heart failure, mice were subjected to AB for 4 weeks to induce heart failure and then injected with AICAR for 4 weeks to continuously activate AMPK. Mice in the control group were injected with saline. As shown in Table 1, in AB-induced failing hearts, AICAR treatment significantly blocked further development of cardiac hypertrophy, as shown by decreases in the heart weight/tibia length ratio (HW/TL) and heart weight/body weight ratio (HW/BW). Moreover, AICAR treatment markedly improved the lung weight/tibia length ratio (LW/TL) and lung weight/body weight ratio (LW/BW), indicating that AMPK activation alleviated pulmonary edema. In addition, echocardiography showed that AICAR treatment could effectively reduce the left ventricular end-diastolic dimension (LVEDd) and left ventricular end-systolic dimension (LVESd) compared with saline treatment, suggesting that AMPK activation can attenuate further cardiac dilation. In addition, cardiac function in mice treated with AICAR was better than that in mice treated with saline based on the ejection fraction (EF) (38.27% compared with 28.63%). Altogether, these findings demonstrated that AMPK blunted the aggravation of heart failure and restored cardiac function in failing hearts.

**Table 1:** Echocardiographic data showed the effects of AICAR on heart failure induced by AB

Parameter	NS + Sham	AICAR + sham	NS + AB	AICAR + AB
**Number**	*n*=8	*n*=8	*n*=8	*n*=8
**BW (g)**	24.38 ± 0.24	25.01 ± 0.13	24.80 ± 0.13	24.68 ± 0.11
**HW/BW (mg/g)**	4.51 ± 0.12	4.40 ± 0.17	10.82 ± 0.86^*^	8.21 ± 0.60^*,†^
**LW/BW (mg/g)**	6.27 ± 0.35	6.03 ± 0.25	12.62 ± 2.06^*^	7.85 ± 0.66^*,†^
**HW/TL (mg/mm)**	6.47 ± 0.29	6.68 ± 0.19	15.70 ± 0.84^*^	12.1 ± 0.93^*,†^
**LW/TL (mg/mm)**	8.92 ± 0.39	9.11 ± 0.19	18.53 ± 3.04^*^	11.60 ± 1.04^*,†^
**LVEDd (mm)**	3.82 ± 0.15	3.86 ± 0.08	5.38 ± 0.29^*^	4.66 ± 0.23^*,†^
**LVEDs (mm)**	2.61 ± 0.14	2.67 ± 0.07	4.65 ± 0.48^*^	3.80 ± 0.37^*,†^
**EF%**	60.42 ± 1.88	59.8 ± 1.58	28.63 ± 2.6^*^	38.27 ± 3.73^*,†^

Data were represented as mean ± S.E.M., *n*=8. ^*^P<0.05 compared with corresponding sham control. ^†^*P*<0.05 compared with NS + AB group.

### AMPK inhibited autophagy in heart failure

**Figure 1:**
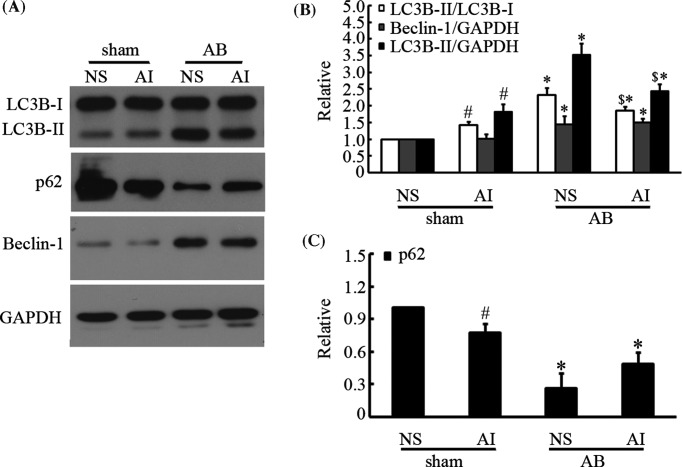
AMPK inhibited autophagy in heart failure Mice were first subjected to AB for 4 weeks to induce chronic heart failure; then, they were treated with intraperitoneal injections of 0.5 mg/g AICAR (AMPK activator) for another 4 weeks. Control mice received an equal volume of normal saline. (**A**–**C**) Representative immunoblots and quantitative analysis of LC3B, beclin-1, and p62 expression in the hearts. *P<0.05 compared with the corresponding sham control group; ^#^*P*<0.05 compared with the NS + sham group; and ^$^*P*<0.05 compared with the NS + AB group (each experiment was repeated four times, *n*=4).

As AMPK has been shown to positively regulate autophagy, we suspected that autophagy was the mechanism involved in the protective effect of AMPK against established heart failure. Therefore, we further detected the effects of AMPK on autophagy in heart failure. Mice were administered AICAR for 4 weeks after AB surgery, when the heart shifted from stable cardiac hypertrophy to heart failure. As shown in Figure 1, compared with that in the saline control group, after AB surgery, 4 weeks of AICAR treatment significantly decreased autophagy in the failing hearts, as manifested by decreased ratios of LC3B-II/LC3B-I and LC3B-II/GAPDH and enhanced p62 expression. In contrast, AICAR notably promoted autophagy in the sham group, which consisted of mice without heart failure. However, the expression level of beclin-1, another autophagy-related protein, was elevated in failing hearts but was not affected by AICAR treatment.

**Figure 2:**
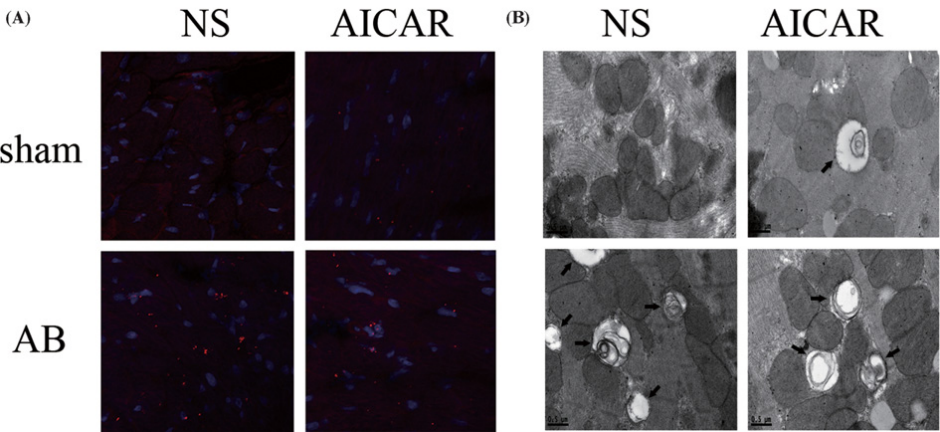
Analysis of autophagy in hearts after AICAR administration (**A**) The number of LC3B dots was elevated in the failing hearts, while treatment with AICAR significantly reduced the number of LC3B dots, suggesting decreased autophagy in the failing hearts following AMPK activation. Moreover, in mice treated with AICAR, remarkable inhibition of autophagosome formation was detected using electron microscopy (**B**). However, in hearts that did not undergo AB surgery, AICAR markedly increased autophagy (A,B).

To further confirm the effect of AMPK activation on autophagy in heart failure, we detected LC3B protein dots in heart tissues using immunofluorescence microscopy. As shown in Figure 2A, the number of LC3B dots was elevated in the failing hearts, while AICAR treatment significantly reduced the number of LC3B dots, suggesting that autophagy was decreased in the failing hearts following AMPK activation. Moreover, in mice treated with AICAR, remarkable inhibition of autophagosome formation was detected using electron microscopy (Figure 2B). In addition, in the hearts without AB surgery, AICAR markedly increased autophagy (Figure 2A,B). Therefore, our results suggested that AMPK has differential effects on autophagy; AMPK activation could reduce autophagy in failing hearts but induce autophagy in non-failing hearts.

### Effects of AMPK on mammalian target of rapamycin signaling in the failing hearts

**Figure 3:**
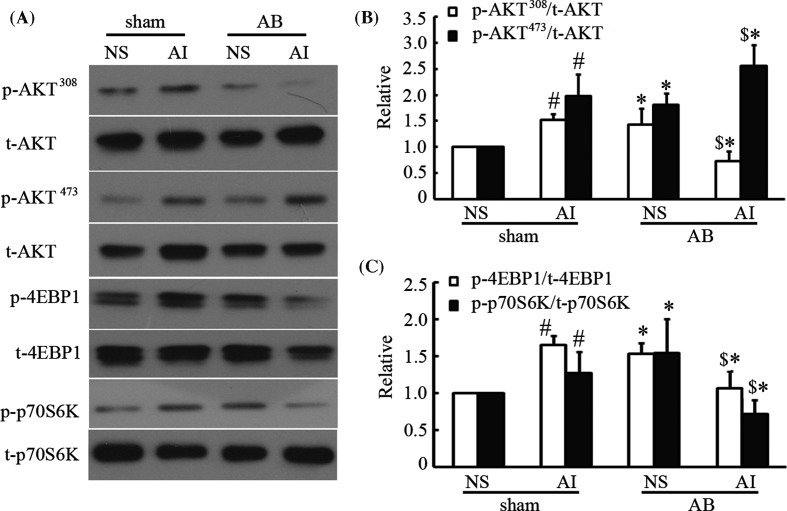
Effects of AMPK on mTOR signaling in the failing hearts Mice were first subjected to AB for 4 weeks to induce chronic heart failure, followed by intraperitoneal injection of 0.5 mg/g AICAR (AMPK activator) for another 4 weeks. Representative immunoblots (**A**) and the related quantitative analysis (**B**,**C**) of phosphorylated and total 4EBP1, p70S6K, and AKT expression in heart failure. *P<0.05 compared with the corresponding sham or vehicle control group; ^#^*P*<0.05 compared with the sham + NS group; ^$^*P*<0.05 compared with the AB + NS group (the blots represent four independent experiments, *n*=4).

mTOR complex (mTORC) exists in two structurally and functionally different forms: mTOR complex 1 (mTORC1) and mTOR complex 2 (mTORC2). S6K and 4EBP1 are the downstream effectors of mTORC1, while AKT (Ser^473^) is the downstream effector of mTORC2. The relationship between mTORC and autophagy has been comprehensively evaluated [6]. In our previous study, we showed that AMPK enhanced autophagy in cardiac hypertrophy due to inhibition of mTORC1 [10]. However, in the present study, we found that AMPK activation could block autophagy in failing hearts. Therefore, we were interested in the role of mTORC in this process. Herein, we detected the activity of mTORC1 and mTORC2 by measuring their downstream effectors, thereby illustrating the roles of mTORC in the inhibition of autophagy by AMPK. As shown in Figure 3A,B, phosphorylation of 4EBP1, p70S6K, and AKT (Ser^473^ and Thr^308^) was increased after AICAR treatment in hearts without AB. However, in the AB-induced failing hearts, AICAR treatment reduced the phosphorylation of 4EBP1 and p70S6K, indicating that AMPK attenuated mTORC1 activation. AKT (Thr^308^) phosphorylation was also down-regulated by AICAR treatment in heart failure, while AKT (Ser^473^), an mTORC2 effector, was activated, suggesting that AMPK activated mTORC2 signaling. Mounting studies have shown that mTORC1 signal inactivation could stimulate autophagy [10,16]; however, the effect of mTORC2 on autophagy is still controvesial [17,18]. In the present study, we observed decreased autophagy and improved cardiac function accompanied by mTORC1 inactivation and mTORC2 activation after AMPK stimulation in the failing hearts. Therefore, we speculated that the cardioprotective effect of AMPK on the failing hearts might be associated with mTORC2 activation, which requires additional studies to verify.

## Discussion

Our previous studies have shown that AMPK can inhibit the development of cardiac hypertrophy and heart failure by promoting autophagy in the pressure overload model. However, in the present study, we found that AMPK activation can preserve cardiac function in heart failure by down-regulating autophagy, which might be associated with mTORC2 activation. Therefore, our findings demonstrated the differential effects of AMPK on autophagy in the process of heart failure.

Basal autophagy is pivotal for maintaining myocardial morphology and cardiac function [16]. Adiponectin might blunt cardiomyocyte hypertrophy and improve cardiac function by stimulating autophagy [19,20]. Improved autophagy by Sirt3 activation also ameliorates myocardial hypertrophy [21]. However, mounting evidence has also shown a positive relationship between heart failure and overactivated autophagy [4,5,22]. Previous studies have demonstrated that AMPK could blunt cardiac hypertrophy by activating autophagy [2,3,10], but overactivated and dysregulated autophagy could also accelerate the development of heart failure [4–6]. Therefore, in the current study, we aimed to explore whether AMPK protected against established heart failure by modulating autophagy. Based on our previous studies and the reports of other groups, AMPK can block further development of heart failure when it is activated during cardiac hypertrophy, indicating that AMPK has already begun to induce autophagy before the onset of heart failure. In the present study, all the mice were first subjected to AB surgery for 4 weeks to induce chronic heart failure and then subjected to 4 weeks of AICAR treatment in the heart failure stage when autophagy was already overactivated. Our results showed that AMPK down-regulates autophagy and restores cardiac function in heart failure. In contrast, AMPK promotes autophagy in the sham group, in which the heart exhibited normal function, suggesting the differential effects of AMPK on autophagy. Therefore, we speculated that AMPK protected cardiac function by up-regulating autophagy in cardiac hypertrophy and by down-regulating the overactivated autophagy in heart failure. Moreover, whether AMPK activates or inhibits autophagy in the process of heart failure development depends on the level of autophagy.

mTOR is the key sensor of nutrient status and exists in two functionally distinct complexes, mTORC1 and mTORC2 [23]. mTORC1, which consists of mTOR, Raptor, mLST8, and PRAS40, usually regulates protein synthesis, cell proliferation, metabolism, and autophagy. mTORC2, which consists of mTOR, rictor, mLST8, Protor (PRR5), and SIN1, typically controls cell survival and polarity [23]. Impaired cells often lose their proliferative potential, lose control over proteostasis and accumulate toxic detritus, which destroy normal cellular metabolism, leading to a phenomenon known as proteotoxic stress. Hypertrophy and senescence of cardiac cells may be significantly suppressed by mTORC1 repression, which is followed by the activation of autophagy, a mechanism by which cells clear a wide range of debris, including toxic protein aggregates that promote the emergence of senescent proinflammatory cell types [24]. mTORC1 is involved in ageing, cardiac hypertrophy, myocardial ischemia, obesity, and metabolic syndrome [6,25–27]. These effects may occur through reducing proteotoxic stressors and decreasing the rate at which hyperinflammatory senescent cellular phenotypes accumulate in tissues [24]. Nevertheless, many pathophysiological effects of mTORC1 still remain unclear. AMPK is a regulator of mTORC1, and the AMPK-mTORC1 pathway is involved in the positive regulation of autophagy [10,24]. Evidence has shown that the AMPK-mTORC1 pathway is at least involved in the regulation of autophagy and energy metabolism in heart failure. In the present study, we found that AMPK inhibits autophagic activity in the failing heart, which is accompanied by the down-regulation of mTORC1. However, there is no optimal mTORC1 inhibitor that would selectively inhibit a specific function of mTORC1 without affecting its other physiological effects. Thus, the effects of mTORC1 on cardiac hypertrophy cannot be completely attributed to its regulation of autophagy. Moreover, a previous study showed that in diabetic heart failure patients, mTORC activity was suppressed, while there was a very little change in AMPK [28]. Therefore, we concluded that the down-regulation of mTORC1 after AMPK activation might not be involved in the regulation of autophagy in heart failure and that other mechanisms, such as energy metabolism, might be involved. The effect of mTORC2 on autophagy is still controvesial [17,18,29]. Knockdown of rictor by shRNA has been shown to induce autophagosome formation in skeletal muscle [18]. In contrast, in cardiac myoblasts, rictor silencing results in suppressed autophagy [17]. In our previous studies, we showed that AMPK inhibited cardiac hypertrophy by promoting autophagy with the involvement of mTORC1 signaling [10]. However, in the present study, we found that AMPK down-regulated autophagic activity in the failing heart, which was accompanied by the activation of mTORC2, suggesting that AMPK ameliorated overactivated autophagy by inducing mTORC2 activation in heart failure. It was also demonstrated that cardiac stromal interaction molecule 1 (STIM1) inhibition promoted heart failure by repressing mTORC2 [30]. In addition, rictor-deficient mice developed cardiac dysfunction 1 week after thoracic aorta constriction (TAC) surgery, indicating that mTORC2 signaling contributes to the maintenance of contractile function in pressure-overloaded mice [31]. Importantly, evidence showed that AMPK could directly promote TORC2 phosphorylation and block TORC2 nuclear accumulation, resulting in attenuation of the gluconeogenic program [29]. Therefore, in our study, we speculated that the activation of AMPK in failing hearts protects against the aggravation of heart failure via the down-regulation of autophagy by mTORC2 activation.

In conclusion, our experiments showed that activation of AMPK in heart failure can ameliorate cardiac dysfunction, potentially by reducing autophagic activity via mTORC2 activation. However, the underlying mechanism acquires further study.

## Abbreviations

AB: aortic banding
AICAR: 5-aminoimidazole-4-carboxamide 1-β-d-ribofuranoside
AMPK: AMP-activated protein kinase
mTOR: mammalian target of rapamycin
mTORC: mTOR complex
PE: phenylephrine
p70S6K: ribosomal protein S6 kinase
4EBP1: 4E-binding protein1
